# Special Issue “Talent Identification and Development in Youth Sports”

**DOI:** 10.3390/sports10120189

**Published:** 2022-11-24

**Authors:** Adam Leigh Kelly, Alberto Lorenzo Calvo, Sara Diana Leal dos Santos, Sergio Lorenzo Jiménez Sáiz

**Affiliations:** 1 Research Centre for Life and Sport Sciences (CLaSS), Department of Sport and Exercise, School of Health Sciences, Birmingham City University, Birmingham B15 3TN, West Midlands, UK; 2 Departamento de Deportes, Facultad de Ciencias de la Actividad Física y del Deporte—INEF, Universidad Politécnica de Madrid, 28040 Madrid, Spain; 3 Research Center in Sports Sciences, Health Sciences and Human Development (CIDESD), University of Trás-os-Montes and Alto Douro, 5000-801 Vila Real, Portugal; 4 Centre for Sport Studies, Universidad Rey Juan Carlos, Fuenlabrada, 28942 Madrid, Spain

## 1. Introduction

We are delighted to share our Special Issue on Talent Identification and Development in Youth Sports. In 2020, the editorial team had several informal discussions about the growing interest from researchers and practitioners in these disciplines and subsequently wanted to create a platform to help advance this field of literature. Following these conversations, we decided to use the Personal Assets Framework [1,2,3] to outline our objectives and the potential research topics for our Special Issue. The overarching aim was to explore how youth’s personal engagement in activities (i.e., the what), quality social dynamics (i.e., the who), and appropriate settings and organisational structures (i.e., the where) can foster immediate, short-term, and long-term developmental outcomes in sport. In doing so, it was hoped that the studies included can inform evidence-based youth sport policies and athlete development programmes. Submissions were encouraged from a diverse range of quantitative and qualitative research methods to examine the current context of talent identification and development in youth sport, as well as reviews to synthesise knowledge within these disciplines.

In light of the articles that have been included within our special issue, we believe our initial aims of progressing the talent identification and development literature have been achieved. We now hope that the research presented can be utilised by key stakeholders (e.g., administrators, coaches, parents, practitioners) and organisational structures (e.g., national governing bodies, professional clubs, recreational teams, youth sport associations) to create more appropriate youth sport settings. To summarise the key messages of the studies in our special issue, this editorial focusses on two fundamental considerations: (a) contextual, and (b) methodological.

## 2. Contextual Considerations

Our Special Issue has supplemented the existing literature to show that contextual factors (e.g., age, gender, nationality, sport popularity, sport type) play an important role in talent identification and development. In particular, the role of birthday and birthplace have been strongly associated with a greater likelihood of being selected into talent pathways and achieving professional status. In relation to ‘relative age effects’, relatively older athletes (i.e., those born near the start of the selection cut-off date) are generally considered to have greater potential and likelihood of being selected. This provides them with more exposure to organised activities (e.g., practice, competition) and resources (e.g., facilities, specialist support), which can facilitate their long-term performance towards adulthood [4]. Conversely, relatively younger athletes (i.e., those born near the end of the selection cut-off date) are less likely to access the same opportunities, which could lead to a negative impact on participation and personal development in the long-term [5,6]. Relative age effects appeared to be the most popular topic in our Special Issue, encompassing twelve studies [7]. For instance, Kelly and colleagues [8] showed relatively older athletes (i.e., those born in the first three months of the year) were up to ten times more likely to be selected into the Basketball England Talent Pathway compared to relatively younger athletes (i.e., those born in the last three months of the selection year); however, findings were more significant in males compared to females. Moreover, McCarthy and colleagues [9] explained the possible mechanisms of relative age effects in rugby union, proposing how challenge was an ever-present feature of all players journeys, especially at the point of transition to senior rugby, whilst psycho-behavioural factors seemed to be a primary mediator of the response to challenge. In addition, Romann and colleagues [10] suggested relative age effects led to inefficient talent selection and an accompanying waste of money when exploring male professional football players’ market values.

Another contextual factor studied that can influence the probability of an athlete participating in high-performance sport is their birthplace (i.e., where the athlete is born and raised). A range of quantitative studies have concluded that athletes born in small cities (<500,000 inhabitants) are more likely to play in professional leagues compared with athletes born in larger cities (>500,000 inhabitants). These ‘birthplace effects’ are likely due to smaller cities being associated with greater development opportunities, effortlessness in the mobility of the athlete, and safety conditions for practice and competition. Within our Special Issue, Maayan and colleagues [11] used a qualitative approach to explore athlete and coach perceptions of birthplace effects from a range of sports, revealing that growing up in cities of small and medium sizes was more beneficial than growing up in towns or cities of other sizes. Most of the coaches they interviewed believed that certain characteristics of the place or city where the athlete grew up (e.g., proximity to sport facilities, access to organised activities) is a significant contributing factor towards talent development.

It is important that the impact of relative age and/or birthplace is not considered to be homogenous. Moving forward, it will be worthwhile to increase the studies of relative age and birthplace to better understand the appropriate climate to develop young athletes in a variety of contexts. Practitioners should be cautious of these influences during talent identification and development, whilst researchers should focus on advancing our understanding of the potential barriers and relationships. This should be conducted both across and within different countries, as the interaction between contextual factors can help explain the trajectory and performance of athletes [12]. Moreover, understanding the connection between relative age and birthplace effects will support researchers and policy makers to design sports systems and policies that help nurture talent more accurately and equitably. However, we already know that producing favourable environments for talent identification and development are highly complex tasks due to the multidimensional nature of development coupled with the continued evolution of sport performance. Thus, researchers are encouraged to design more longitudinal, multidimensional, and prospective studies in order to capture the trajectories of youth athletes in diverse youth sports settings.

Another approach that has received growing attention, as highlighted by Sæther and colleagues [13], is the ‘talent transfer’. This refers to the intention of a talented athlete choosing to invest in other sports, such as transferring athletes from donor sports into target sports or the transition from summer (e.g., kayaking or rowing) to winter (e.g., cross-country skiing) sports. Thus, it is important to deepen our knowledge to understand: (a) the range of elements (e.g., technical, tactical, physical, psychological, social) that act as facilitators to help athletes successfully transfer from one sport to another, (b) whether there are donor sports that are more suitable for recruitment into other sports, and (c) the effectiveness of existing talent transfer programmes. Considering this, practitioners should be aware that there are many critical determinants to talent identification and development; therefore, the incorporation of multi-method research approaches across and within different sporting and sociocultural contexts could be an avenue for future research.

## 3. Methodological Considerations

The editorial team made a concerted effort to recruit diverse authors who could support this research topic, and we are incredibly grateful to all the researchers for their strong contributions. We also aimed to capture varied methodological approaches to ensure the Special Issue offered a unique contribution to the field of talent identification and development in youth sport. The 34 articles (31 empirical studies and 3 reviews) that appear were penned by 128 authors (several whom appear more than once), many of which are internationally recognised scholars in this field. These authors represent universities or sport institutions (i.e., professional sport teams or national governing bodies) from across 20 countries. Owing to our approach, it is not surprising that 17 sports are studied within this Special Issue, which includes 13 studies comprising female participants. While soccer remains at the fore of the evidence-base with twelve articles, it is pleasing to see more under-researched talent development contexts also being considered (e.g., [14]). Ultimately, we believe that the diverse authorship, contexts, and samples have resulted in a unique Special Issue, which has significantly advanced the field through its various approaches that should facilitate thoughtful discussion about talent identification and development.

A range of quantitative (e.g., Bayesian machine learning, chi-square, coding, discriminant analyses, mixed multilevel logistic models) and qualitative (e.g., abductive hierarchical content analysis, ethnography, inductive approach, realist evaluation approach, reflexive thematic analysis) data collection and analysis methods were used throughout this Special Issue. Not only do these approaches provide novel insights into talent identification and development, but they also offer researchers the opportunity to replicate studies in different settings. One rapidly emerging quantitative analysis approach that was used twice in this Special Issue [15,16] is machine learning. For instance, Owen and colleagues [16] used Bayesian machine learning to create predictive models for selected and non-selected Welsh male U16 and U18 rugby players. Whilst they showed their physiological and psychosocial models correctly classified 67.5% and 62.3% of all players, respectively, they also provided a unique method to explore selection into talent pathways that may be replicable to other researchers in the future.

From a qualitative perspective, Lara-Bercial and McKenna [17,18] produced a two-paper series using a season long ethnography of a youth performance sport club based on a novel realist evaluation approach. In the first study [17], the authors detailed the perceptions of club stakeholders to build a set of programme theories, with the resulting network of outcomes (i.e., self, emotional, social, moral, and cognitive) and generative mechanisms (i.e., the attention factory, the greenhouse for growth, the personal boost, and the real-life simulator) providing a nuanced understanding of stakeholders’ views and experiences. In the second study [18], the lead author spent a full season in the club, whereby the collection of context–mechanism–outcome networks (CMONs) described in the first study was used to guide the researcher during their immersive period. Such qualitative approaches offer a different lens to those typically incorporated in talent identification and development, which may shed light on findings that may not be captured during traditional approaches.

It is also important to consider how articles can be grounded or discussed through relevant models or theories (e.g., [19,20]). As a novel example, Kelly and colleagues [21] used the Personal Assets Framework to explain the immediate, short-term, and long-term developmental outcomes due to relative age effects in English male cricket. Indeed, using such an approach helps give the study a well-defined and proven basis of argument or phenomena, offers an explanation of the study’s significance and validity, and shows where the researcher intends to fill in gaps of knowledge and practice. Moving forward, we lend our guidance using Barraclough and colleagues’ [22] narrative review, which summarised methodological approaches to talent identification in team sports. The authors highlight the benefits of longitudinal, multidisciplinary, and ecologically valid research designs. Specifically, they outline three key areas for consideration for future research: (a) the timespan of the research design, (b) the use of monodisciplinary or multidisciplinary variables, and (c) the fidelity of the methodological approaches to the assessment of talent. One final methodological consideration, as highlighted by Mosher and colleagues [23], is the growing issue of the language that is used in sport science literature. Following their investigation into early specialisers, the authors underscored that the main rationale related to their study was the lack of terminological and conceptual concreteness related to this topic. As such, it is necessary to improve and develop new methodologies and concrete definitions that allow us to access much more reliable and externally valid data.

## 4. Future Directions

Grounded on contemporary talent identification and development knowledge, this Special Issue provides useful insights to drive future advances in research and practice. Currently, gaps arise when considering the limitations of unidimensional assessment models (i.e., static and isolated variables) to identify talent and capture the dynamic nature of sports. Thus, it is necessary that future studies conduct more valid, reliable, and multidisciplinary assessment procedures in an effort to more accurately and resourcefully identify and develop talented youth athletes (e.g., [24,25]). Furthermore, several studies included in our Special Issue suggest that future directions should consider longitudinal tracking of interacting factors [26], such as the quantity, quality, and type of practice during sport participation [27], as well as monitoring biological maturation [28,29], physical performance [30,31], technical skills [32], and psychological profiles [33,34]. Evaluating how stakeholders communicate and implement these theoretical findings into applied settings will also be important for future research to ensure they are adequately deployed (e.g., [35,36,37]). Finally, it is vital for future research to consider the contextual and methodological implications of COVID-19 in youth sport (see [38] for an overview), since the immediate, short-term, and the long-term impact of a global pandemic on the identification and development of young athletes remains relatively unknown and could have enduring consequences [39].

## 5. Summary

The main purpose of research is to enhance real-life settings by advancing knowledge through the development of scientific theories, concepts, and ideas. In relation to this Special Issue, it is hoped that the articles presented can be utilised by key stakeholders and organisational structures to create more appropriate youth sport settings. It was a pleasure to assemble this resource and is hoped the contextual and methodological considerations presented throughout this editorial provide researchers and practitioners with a range of thought-provoking concepts for their respective agendas. Thank you to all the contributing authors and reviewers, without whom, this research topic would not be possible.
